# Pharmacokinetic properties of intramuscular *versus* oral syrup paracetamol in *Plasmodium falciparum* malaria

**DOI:** 10.1186/s12936-016-1283-9

**Published:** 2016-04-27

**Authors:** Thanaporn Wattanakul, Pramote Teerapong, Katherine Plewes, Paul N. Newton, Wirongrong Chierakul, Kamolrat Silamut, Kesinee Chotivanich, Ronnatrai Ruengweerayut, Nicholas J. White, Arjen M. Dondorp, Joel Tarning

**Affiliations:** Mahidol-Oxford Tropical Medicine Research Unit, Faculty of Tropical Medicine, Mahidol University, Bangkok, Thailand; Nuffield Department of Medicine, Centre for Tropical Medicine and Global Health, University of Oxford, Oxford, UK; Primary Care Unit, Siriraj Hospital, Mahidol University, Bangkok, Thailand; Lao-Oxford-Mahosot Hospital-Wellcome Trust Research Unit, Mahosot Hospital, Vientiane, Lao People’s Democratic Republic; Department of Medicine, Mae Sot Hospital, Mae Sot, Thailand

**Keywords:** Paracetamol, Pharmacokinetics, Falciparum malaria, Intramuscular, Randomized crossover trial, Antipyretic, NONMEM

## Abstract

**Background:**

Fever is an inherent symptom of malaria in both adults and children. Paracetamol (acetaminophen) is the recommended antipyretic as it is inexpensive, widely available and has a good safety profile, but patients may not be able to take the oral drug reliably. A comparison between the pharmacokinetics of oral syrup and intramuscular paracetamol given to patients with acute falciparum malaria and high body temperature was performed.

**Methods:**

A randomized, open-label, two-treatment, crossover, pharmacokinetic study of paracetamol dosed orally and intramuscularly was conducted. Twenty-one adult patients with uncomplicated falciparum malaria were randomized to receive a single 600 mg dose of paracetamol either as syrup or intramuscular injection on day 0 followed by a single dose administered by the alternative route on day 1. Paracetamol plasma concentrations were quantified frequently and modelled simultaneously using nonlinear mixed-effects modelling. The final population pharmacokinetic model was used for dose optimization simulations. Relationships between paracetamol concentrations with temperature and parasite half-life were investigated using linear and non-linear regression analyses.

**Results:**

The population pharmacokinetic properties of paracetamol were best described by a two-compartment disposition model, with zero-order and first-order absorption for intramuscular and oral syrup administration, respectively. The relative bioavailability of oral syrup was 84.4 % (95 % CI 68.2–95.1 %) compared to intramuscular administration. Dosing simulations showed that 1000 mg of intramuscular or oral syrup administered six-hourly reached therapeutic steady state concentrations for antipyresis, but more favourable concentration–time profiles were achieved with a loading dose of 1500 mg, followed by a 1000 mg maintenance dose. This ensured that maximum therapeutic concentrations were reached rapidly during the first 6 h. No significant relationships between paracetamol concentrations and temperature or parasite half-life were found.

**Conclusions:**

Paracetamol plasma concentrations after oral syrup and intramuscular administration in patients with acute falciparum malaria were described successfully by a two-compartment disposition model. Relative oral bioavailability compared to intramuscular dosing was estimated as 84.4 % (95 % CI 68.2–95.1 %). Dosing simulations showed that a loading dose followed by six-hourly dosing intervals reduced the time delay to reach therapeutic drug levels after both routes of administration. The safety and efficacy of loading dose paracetamol antipyretic regimens now needs to be established in larger studies.

## Background

The clinical features of all human malarias start non-specifically with influenza-like symptoms, including fever. Rising temperatures initially cause shivering, mild chills, worsening headaches, malaise, and loss of appetite. Fever in malaria is initially usually irregular. In untreated infection, the fever in *Plasmodium falciparum* can regularize to a 2-day cycle (tertian malaria), although this is more variable than in infections with *Plasmodium vivax*. Before treatment in synchronous infections classic periodic ‘paroxysms’ typically occur every 2 days (three in *P*. *malariae* infections) characterized by an abrupt steeply rising temperature to >39 °C with intense headache, uncomfortable ‘cold chills’ with peripheral vasoconstriction, and often frank rigors with shaking limbs and teeth chattering [1]. These paroxysms are more likely with relapses, and although firmly established in the history and nomenclature of malaria, they are rarely observed today in the era of prompt and effective anti-malarial drug treatment. In adult patients with severe *P. falciparum* malaria, 60 % present with fever ≥38 °C. There is increased mortality in hyperpyrexic patients (>40.5 °C) [2]. Fever in malaria is associated with anorexia, nausea and vomiting, which exacerbate dehydration due to insensible losses. Fever may also increase sequestration of infected red blood cells and thus potentially contribute to clinical deterioration in severe malaria patients. The increased metabolic rate associated with fever exacerbates anaerobic glycolysis in vital organs affected by microvascular obstruction [3]. The World Health Organization (WHO) recommends giving regular paracetamol every 6 h if core temperatures exceed 38.5 °C [4, 5]. Studies without pharmacokinetic assessments have shown conflicting results regarding the effects of paracetamol on fever and parasite clearance in patients with uncomplicated malaria [6–8]. In practice, achieving therapeutic (antipyretic) plasma concentrations may be compromised, since paracetamol is often given irregularly at low doses and patients who are nauseated or unconscious will be unable to take paracetamol orally. Paracetamol given orally or via a nasogastric tube is subjected to approximately 20 % first-pass metabolism [9–11]. Pharmacokinetic studies show that higher dosing is required to achieve therapeutic serum concentrations when paracetamol is given by the rectal route [12–14]. Due to the practical obstacles of delivering adequate suppository doses to adults, the majority of patients are under-dosed by this route [15].

In many malaria-endemic countries intramuscular paracetamol is used widely as an antipyretic in patients with malaria, particularly in those unable to take oral medication. However, relative bioavailability and antipyretic efficacy of intramuscular paracetamol in patients with malaria have never been investigated. A study of intramuscular paracetamol in children undergoing minor surgery showed that intramuscular paracetamol achieved higher drug levels compared to suppositories [16]. However, in sick patients with poor muscle perfusion the relative bioavailability of intramuscular paracetamol could be reduced. To determine the preferred route of administration, a randomized, crossover, pharmacokinetic comparison of paracetamol given by the intramuscular route *versus* oral/nasogastric tube route in patients with acute falciparum malaria and high fever was conducted.

## Methods

### Study design and patients

The study was a randomized, open-label, two-treatment, crossover study conducted at Mae Sot Hospital, Tak, Thailand from May to June 2001. Malaria transmission in this area is low and seasonal with peak transmission during the rainy season from May to August. All age groups are affected. Consecutive non-pregnant adult (≥15 years) patients admitted with slide-confirmed, uncomplicated falciparum malaria were enrolled if they had an aural temperature >38 °C, required oral or intramuscular paracetamol and were willing to provide informed consent. Patients were excluded if paracetamol had been taken within the previous 12 h, if there was any contraindication to paracetamol or requirement for interacting drugs. Criteria for uncomplicated malaria included the absence of all the following: coma (Glasgow Coma Score <11), shock (systolic blood pressure (SBP) <80 mmHg with cool extremities), severe anaemia (haematocrit <20 % plus parasitaemia >100,000/µl), severe jaundice (total bilirubin >2.5 mg/dl plus parasitaemia >100,000/µl), hyperparasitaemia (peripheral asexual stage parasitaemia >10 %), acidosis (venous bicarbonate <15 mmol/l), hyperlactataemia (venous lactate >4 mmol/l), hypoglycaemia (blood glucose <40 mg/dl), and renal failure (serum creatinine >3 mg/dl with urine <400 ml/24 h). Informed written consent was obtained from each patient before randomization and study procedures. The Ministry of Public Health, Royal Government of Thailand granted ethical approval for the study. This study was conducted in 2001 prior to trial registration and CONSORT statement recommendations.

### Drug administration

This was a two-treatment, crossover study of oral and intramuscular paracetamol. Study participants were randomly assigned to receive on the first day of enrolment, either a single dose of 600 mg paracetamol syrup suspension (oral or via nasogastric tube) (Tylenol®; Janssen), followed by 100 ml of water (Group 1), or a single dose of 600 mg (300 mg/2 ml) intramuscular paracetamol divided in two 2-ml doses into the anterior thigh (Group 2) (Partamol®; Atlantic; per ml: 150 mg paracetamol, 0.4 ml tetraglycol, 0.02 ml benzyl alcohol, 20 mg sodium benzoate in sterile water). After a period of 24 h, the patients received paracetamol by the alternate route. A computerized, randomization schedule generated treatment allocations that were implemented by drawing an individual pre-prepared, sealed and sequentially numbered opaque envelope for each enrolled participant. As an open-label study, blinding of investigators and patients was not applicable. However, the randomization procedure allowed for adequate drug allocation concealment before envelopes were opened. All laboratory investigations were performed without knowledge of the treatment allocation.

Study participants whose aural temperature exceeded 40 °C despite the study dose of paracetamol, received additional tepid sponging and a bedside fan. If the aural temperature exceeded 40 °C after 6 h from the first dose of paracetamol, further doses were administered to a daily maximum of 4 g. The dosage and time of each additional paracetamol dose were recorded.

Anti-malarial treatment consisted of intravenous artesunate (Guilin No. 2 Pharmaceuticals, China) 2.4 mg/kg on admission, followed by 1.2 mg/kg every 12 h for the first 24 h of admission, followed by daily oral artesunate (2 mg/kg; Guilin No. 2 Pharmaceuticals, China) combined with doxycycline (4 mg/kg per day in two doses; Vibramycin, Pfizer) for a total of 7 days. At the time of the study, this anti-malarial regimen was proven to be effective in this area of emerging highly resistant *P. falciparum*. All participants were managed according to WHO treatment guidelines [17].

### Study assessments and investigations

On enrolment, a full medical history and examination, including aural temperature, height and weight, were performed. Venous blood was collected for baseline venous blood biochemistry, including creatinine, blood urea nitrogen (BUN), bilirubin (total and indirect), alanine transaminase (ALT), aspartate transaminase (AST) and alkaline phosphatase (ALP), as well as haematocrit and peripheral blood parasitaemia. Parasite counts were repeated six-hourly until parasite clearance, defined as two consecutive negative blood smears. Lithium heparin plasma samples for paracetamol concentration measurement were collected on enrolment (pre-dose), and then at 0.5, 1.0, 1.5, 2, 3, 4, 6, 8, 10 and 12 h post-dose. Aural temperature was monitored and recorded at each blood sampling time point. Plasma samples were processed and stored at −80 °C for further analysis in Bangkok, Thailand. Paracetamol plasma concentrations were quantified using high-performance liquid chromatography, as previously described [9, 10].

### Population pharmacokinetic and pharmacodynamic analysis

Paracetamol plasma concentrations after intramuscular and oral administration were transformed into their natural logarithms and modelled simultaneously using NONMEM, version 7.2 (Icon Development Solution, Ellicott City, MD, USA). Model diagnostics and automation were performed using Xpose version 4.0 [18], Pirana [19], and Pearl-speaks-NONMEM (PsN; version 3.5.3) [20]. The first-order conditional estimation method with interaction was used throughout the model development. The difference in objective function value (ΔOFV; calculated by NONMEM as proportional to −2× the log-likelihood of data) was used as a statistical criterion for discrimination of hierarchical models. ΔOFV of >3.84 and >10.83 were considered statistically significant at *p* value of <0.05 and <0.001, respectively, with one degree of freedom. Goodness-of-fit and simulation-based diagnostics were used for assessing the descriptive and predictive performances of the model. There were no observed data below the limit of quantification reported in this dataset.

One-, two-, and three-compartment disposition models were evaluated to assess the distribution of paracetamol into “shallow” and “deep” body compartments. A one-compartment disposition model assumes that the whole body is a single unit in which the drug is distributed instantaneously. Two- and three-compartment disposition models are represented by central and peripheral compartments with different distributional rate constants between compartments to describe multi-phasic concentration–time profiles. The disposition model that best described the observed concentration–time profile of the drug was used as the structural model for further investigation. Different absorption models were investigated to describe the absorption process after intramuscular and oral administration, including zero-order absorption, first-order absorption with and without lag time, and a flexible transit-absorption model [21]. Zero-order absorption represents a constant amount of paracetamol absorbed per unit of time, whereas first-order absorption is characterized by a constant absorption rate of paracetamol (i.e. the amount absorbed per unit of time is dependent on the amount available to be absorbed). A transit-absorption model is more physiological representation of the absorption process in which the drug moves through a number of hypothetical transit compartments before being absorbed with a first-order rate constant into the systemic circulation. For patients who had measurable pre-dose concentrations of paracetamol, baseline estimation was implemented in these patients. Inter-individual variability was introduced exponentially (Eq. 1).

1 $$\theta_{i} = \theta \times \exp \;(\eta_{i,\theta } )$$

where $\theta_{i}$ is the individual ‘*i*’ parameter estimate, $\theta$ is the population mean parameter estimate, and $\eta_{i,\theta }$ is the inter-individual variability with zero mean and variance. Variability components with an estimated coefficient of variation (%CV) of less than 1 % were fixed to zero. The residual unexplained variability was assumed to be additive on a logarithmic scale, essentially equivalent to an exponential error on an arithmetic scale.

Body weight was evaluated as an allometric function on all clearance and volume of distribution parameters (Eq. 2).

2 $$\theta_{i} = \theta \times \exp \;(\eta_{i,\theta } ) \times \left( {\frac{{{\text{BW}}_{i} }}{{{\text{BW}}_{median} }}} \right)^{\text{n}}$$

where $BW_{i}$ represents individual body weight and $BW_{median}$ represents median body weight of the study population and n was set to be equal to 0.75 and 1 for all clearance parameters and volume of distribution parameters, respectively.

In order to identify the influence of demographic patient characteristics on pharmacokinetic parameters that may reduce the unexplained inter-individual variability in the model, the standard stepwise forward inclusion and stepwise backward deletion approach (*P* value <0.05 and <0.001 for forward and backward step, respectively) was performed. The following admission patient characteristics were evaluated as covariates by this approach: age (years), AST (U/l), ALT (U/l), bilirubin (mg/dl), BUN (mg/dl), creatinine (mg/dl), gender, haemoglobin (g/dl), parasite count (parasites/μl), systolic blood pressure (mmHg), and temperature (°C).

Bootstrapping (n = 1000), stratified for administration route, was used to assess the robustness of pharmacokinetic parameter estimates from the final model. Numerical and visual predictive checks (n = 2000) were used to evaluate the predictive performance of the final model.

Final pharmacokinetic population parameter estimates from NONMEM were used to simulate different dosing scenarios in Berkeley Madonna [22]. The therapeutic target level of paracetamol was assumed to be 10–20 mg/l [23, 24]. Different dosage regimens were investigated based on a maximum dose of 4 g paracetamol per day and the available paracetamol products (i.e., 500 mg oral tablet and 300 mg/2 ml for injection).

To assess the pharmacodynamic effect of paracetamol on fever control, the relationship between maximal paracetamol concentration (C_MAX_) and temperature reduction 2 h post-dose (Δtemperature _0−2h_, AUC_temperature >37.5 °C at 0–2h_) was assessed using ordinary linear regression. This temperature endpoint was chosen because the maximum median temperature drop occurred at 2 h. The effect of paracetamol on parasite clearance half-life was assessed by two methods: first, by linear regression to assess the relationship between paracetamol C_MAX_ and parasite clearance half-life; second, using a simplified population parasite clearance model based on all available parasite count data, i.e., an estimated baseline parasite biomass and an estimated first-order parasite clearance rate, including inter-individual variability on both parameters. In order to investigate the relationship between plasma concentration of paracetamol and the relative change in parasite clearance rate, individually predicted paracetamol concentrations were derived from the final pharmacokinetic model and then evaluated in the model as a time-varying covariate on the parasite clearance rate using a linear relationship. An indirect paracetamol concentration-effect model (i.e., using a hypothetical effect-compartment) was also assessed. Linear regression analyses were performed using Prism version 6.01 (GraphPad Software, USA). The significance level was defined at *P*  = 0.05.

### Statistical analysis

Non-normally distributed data were compared by the Mann-U Whitney test. Parasitaemia was log-transformed to normality and compared using the Student’s *t*-test. Categorical variables were compared using Fisher’s exact test. Parasite clearance half-life was calculated for the pharmacodynamic analysis using the Worldwide Antimalarial Resistance Network (WWARN) parasite clearance estimator [25]. Secondary pharmacokinetic parameters in both groups (i.e., intramuscular and oral administration) were compared using the Wilcoxon matched-pairs signed rank test. Statistical software used were STATA12.1 (STATA, USA) and Prism 6 (Graphpad Software, USA).

## Results

### Baseline characteristics

**Fig. 1:**
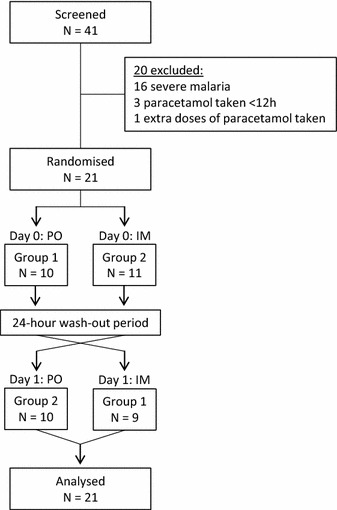
Patient flow diagram. After enrolment to the studies, patients admitted to Mae Sot Hospital had blood collected prior to paracetamol administration followed by timed blood collections. Group 1 received oral syrup paracetamol (PO) on day 0 then intramuscular paracetamol (IM) on day 1; Group 2 received intramuscular paracetamol on day 0 then oral syrup paracetamol on day 1. One patient in Group 1 did not receive the intramuscular dose of paracetamol on day 1 due to self-discharge from the hospital; one patient from Group 2 did not receive oral paracetamol on day 1 as the patient received multiple doses of paracetamol during the study period. All patients were included in the pharmacokinetic analysis

Twenty-one adults with acute falciparum malaria were included in this analysis (Fig. 1). Admission temperature ranged from 38.1 to 41.2 °C. Baseline characteristics are shown in Table 1. Of the 21 patients, seven received extra doses of paracetamol during the 2-day study period. One patient in Group 1 withdrew from the study 12 h after the initial oral dose and did not receive the intramuscular dose on the second day. One patient in Group 2 was given extra oral doses starting 6 h after the initial intramuscular study dose for high fever (>39 °C) and did not receive the scheduled oral study dose the second day. Participants in Group 2 were older, weighed less and had increased ALT and ALP concentrations compared to Group 1 (Table 1). One patient in Group 2 was subsequently diagnosed as having severe malaria based on a total bilirubin >3 mg/dl with a parasitaemia >100,000/μl. Concomitant drugs taken by the patients included dimenhydrinate (n = 2), metoclopramide (n = 4) and domperidone (n = 1). No patient reported excessive alcohol intake.

**Table 1:** Baseline characteristics stratified by treatment group

Variable	Total	Group 1	Group 2
	(n = 21)	(n = 10)	(n = 11)
Age (years)	25 (22–37; 15,54)	23 (20–30; 15,37)	33 (22–52; 20–54)
Males (%)^a^	19 (90)	9 (90)	10 (91)
Weight (kg)	58 (55–63; 47,70)	60 (58–65; 55,70)	55 (50–60; 47,70)
Temperature (°C)	39.6 (39.0–40.5; 38.1,41.2)	40.1 (38.2–40.5; 38.1,41.2)	39.4 (39.0–40.5, 38.6,40.8)
Heart rate (beats/min)	100 (92–115; 80,122)	109 (95–120; 92,122)	94 (90–104; 80,120)
Parasitaemia (parasites/μl)^b^	47,573 (19,769–114,477)	46,793 (13,117–166,929)	48,293 (11,436–203,926)
Haematocrit (%)	44 (38–46; 22,54)	44 (36–45; 34,51)	45 (41–48; 22,54)
Creatinine (μmol/l)	101 (87–112; 70,136)	99 (83–113; 70,131)	105 (92–111; 81,136)
BUN (mg/dl)	16 (15–22; 12,34)	15 (14–21; 13,34)	16 (15–22; 12,29)
AST (IU/l)	40 (27–49; 14,132)	32 (26–42; 14,67)	45 (30–52; 25,132)
ALT (IU/l)	16 (10–28; 9,40)	11 (10–15; 9,28)	26 (16–36; 9,40)
ALP (IU/l)	88 (69–105; 54,311)	72 (63–96; 54,218)	98 (87–116; 70,311)
Total bilirubin (mg/dl)	1.6 (1.2–2.2; 0.6,6.9)	1.9 (1.5–2.4, 1.0,2.7)	1.3 (0.9–1.6; 0.6,6.9)
Indirect bilirubin (mg/dl)	0.4 (0.3–0.5; 0.2,2.4)	0.5 (0.4–0.6; 0.2,0.7)	0.4 (0.3–0.4, 0.2,2.4)

All values are compared using the Mann U Whitney test and reported as median (IQR; range), unless otherwise specified

*BUN* blood urea nitrogen, *AST* aspartate transaminase, *ALT* alanine transaminase, *ALP* alkaline phosphatase

^a^Reported as number (%) and compared using the Fisher’s exact test

^b^Reported as geometric mean (95 % CI) and compared using the Student’s *t*-test. *Group 1* Oral syrup paracetamol on day 0 then intramuscular paracetamol on day 1; *Group 2* Intramuscular paracetamol on day 0 then oral syrup paracetamol on day 1

### Pharmacokinetics

Of the 21 patients, 363 plasma paracetamol concentrations were included in the pharmacokinetic analysis. Paracetamol concentration–time data were well described by a two-compartment disposition model. Adding an additional disposition compartment improved the model fit *P* < 0.05), but not goodness-of-fit plots and visual predictive checks. However, the main driver of this significant improvement in model fit was one patient, resulting in a non-significant (*P* > 0.05) improvement in model fit when this patient was omitted. Furthermore, the terminal half-life estimated from the three-compartment disposition model was unrealistically long (median terminal half-life of 600 h) compared to extensive previous investigations (1.5–2.5 h) [26]. Thus, the two-compartment disposition model was carried forward. Zero-order absorption for intramuscular administration and first-order absorption for oral administration best described the absorption phase of paracetamol compared to all other absorption models (ΔOFV > −62.1).

Allometric scaling of pharmacokinetic parameters did not improve model fit significantly. Thus, body weight was not incorporated into the final model. The stepwise covariate search showed no significant relationships in this population. Inter-individual variability in the duration of zero-order absorption and apparent of volume of distribution were less than 1 % and fixed to zero, without affecting the OFV. Parameter estimates from the final paracetamol pharmacokinetic model are shown in Tables 2 and 3.

**Table 2:** Parameter estimates of the final population pharmacokinetic model of paracetamol in patients with falciparum malaria

Parameter	Population estimate^a^ (%RSE)^b^	95 % CI^b^	IIV (%CV)^a^ (%RSE)^b^	95 % CI^b^
F_IM_	1 fixed	–	–	–
F_PO_	0.844 (8.4)	0.682–0.951	287 (49.1)	76.5–1038
DUR_IM_ (h)	0.689 (6.2)	0.621–0.784	–	–
k_a,PO_ (h^−1^)	4.15 (44.5)	1.95–9.73	232 (49.7)	39.9–574
CL (l/h)	10.7 (16.9)	7.35–14.7	81.8 (69.7)	24.4–164
V_C_ (l)	45.5 (8.5)	36.7–51.5	–	–
Q (l/h)	10.3 (36.8)	4.80–20.1	77.4 (44.0)	21.1–105
V_P_ (l)	11.3 (42.7)	5.01–29.0	428 (46.5)	112–2351
σ	0.376 (7.8)	0.316–0.436	–	–

*F*_*IM*_ bioavailability after intramuscular administration, *F*_*PO*_ relative bioavailability after oral syrup administration, *DUR*_*IM*_ duration of zero-order absorption after intramuscular administration, *k*_*a,PO*_ absorption rate constant after oral syrup administration, *CL* apparent elimination clearance, *V*_*C*_ apparent volume of distribution of the central compartment, *Q* inter-compartment clearance, *V*_*P*_ apparent volume of distribution of the peripheral compartment, *σ* variance of the residual variability

^a^Population mean values and inter-individual variability (IIV) estimated by NONMEM. The coefficient of variation (% CV) for IIV was calculated as $100 \times \sqrt {\exp ({\text{estimate}}){ - }1}$

^b^The relative standard error (%RSE) was calculated as $100 \times \left( {\frac{\text{SD}}{{{\text{Mean}}\;{\text{value}}}}} \right)$ from the non-parametric bootstrap results (n = 1000). The 95 % confidence interval (95 % CI) is presented as the 2.5–97.5 percentiles of the bootstrap estimates

**Table 3:** Secondary parameters of paracetamol after intramuscular and oral syrup administration in patients with falciparum malaria

Secondary parameters	Intramuscular administration	Oral administration	*P* value
C_MAX_ (mg/l)	11.4 (10.8–11.8)	8.52 (7.42–9.55)	<0.0001
T_MAX_ (h)	0.689 (fixed)	0.705 (0.577–1.00)	0.442
t_1/2_ (h)	3.18 (2.67–4.30)	3.03 (2.07–3.53)	0.196
AUC_0–12_ (h × mg/l)	37.9 (27.5–44.9)	31.6 (27.0–39.3)	0.229

*C*_*MAX*_ maximum concentration, *T*_*MAX*_ time to reach maximum concentration, *t*_*1/2*_ terminal half-life, *AUC*_*0–12*_ area under the concentration–time curve from time 0 to 12 h

Secondary parameters were calculated as the median and range of the empirical Bayes estimates. The *P* values were calculated with the Wilcoxon matched-pairs signed rank test

**Fig. 2:**
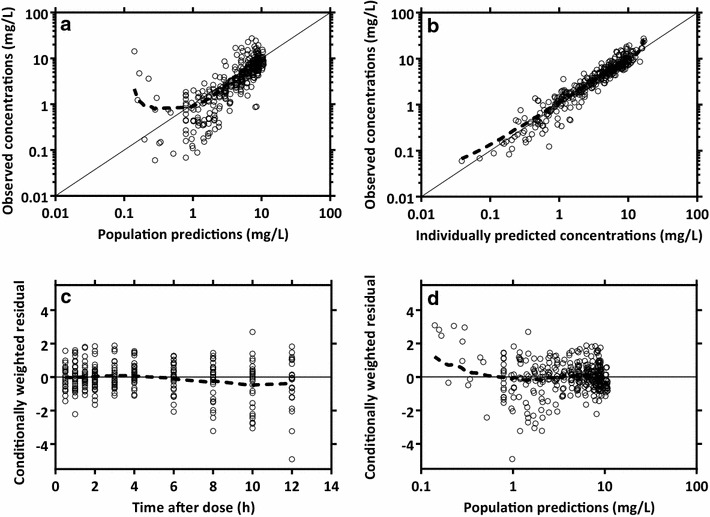
Goodness-of-fit diagnostics of the final population pharmacokinetic model for paracetamol in patients with falciparum malaria. **a** observed concentrations plotted against population predictions, **b** observed concentrations plotted against individually predicted concentrations, **c** conditionally weighted residual plotted against time after dose, and **d** conditionally weighted residual plotted against population predictions. Observations are represented by *black circles*, *solid black lines* represents the line of identity or zero line, and the local polynomial regression fitting for all observations is represented by the *dashed black line*. The observed paracetamol concentrations, population predictions and individual predictions were transformed into their logarithms (base 10)

**Fig. 3:**
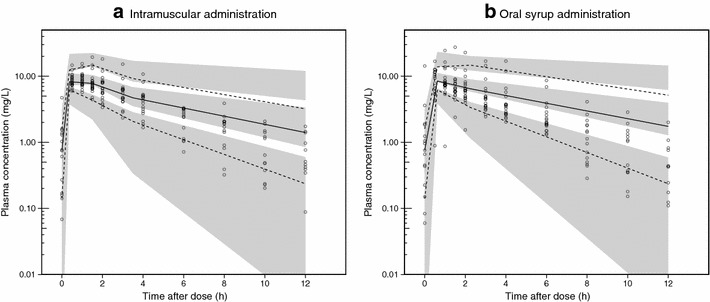
Visual predictive check of the final population pharmacokinetic model for paracetamol in patients with falciparum malaria stratified by route of drug administration. **a** Intramuscular administration and **b** oral syrup administration.* Open circles* observed data points; *solid lines* 5th, 50th and 95th percentiles of the observed data; *shaded area* 95 % confidence intervals of the simulated 5th, 50th and 95th percentiles (n = 2000)

The final paracetamol pharmacokinetic model showed satisfactory goodness-of-fit diagnostics (Fig. 2) and visual predictive performance (Fig. 3). Eta-shrinkage was less than 30 % except for inter-individual variability of inter-compartmental clearance and absorption rate constant (54.9 and 35.8 %, respectively). The numerical predictive check after intramuscular administration resulted in 1.11 % (95 % CI 0.556–12.2 %) and 2.22 % (95 % CI 1.11–10.6 %) of observations below and above the simulated 90 % prediction interval, respectively. The numerical predictive check after oral administration resulted in 1.09 % (95 % CI 0–12.6 %) and 6.56 % (95 % CI 1.09–10.9 %) of observations below and above the simulated 90 % prediction interval, respectively.

**Fig. 4:**
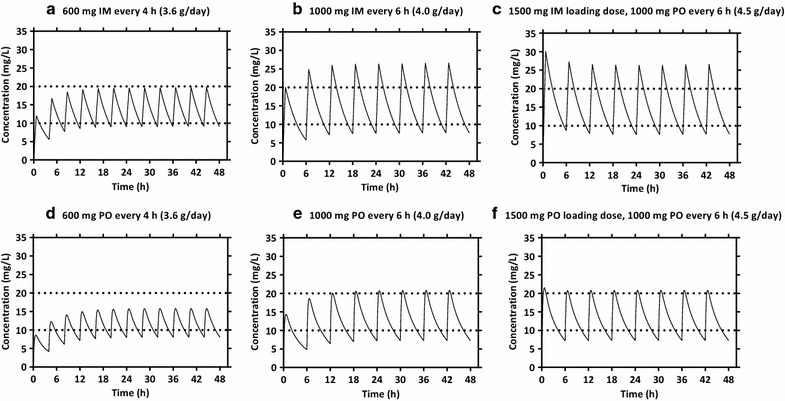
Simulations from the final population pharmacokinetic model for paracetamol. The *lower* and *upper dashed lines* represent the therapeutic plasma concentration range of paracetamol for fever control (10–20 mg/l) [23, 24]. *Upper panel* population mean plasma concentration–time profiles after intramuscular (IM) administration of **a** study dosing regimen; 600 mg every 4 h, **b** normal dosing regimen; 1000 mg every 6 h and **c** modified dosing regimen; 1500 mg loading dose followed by 1000 mg every 6 h. *Lower panel* population mean plasma concentration–time profiles after oral syrup (PO) administration of **d** study dosing regimen; 600 mg every 4 h, **e** normal dosing regimen; 1000 mg every 6 h and **f** modified dosing regimen; 1500 loading followed by 1000 mg every 6 h

Two simulated dosing regimens of 1000 mg intramuscular and oral syrup every 6 h (Fig. 4) achieved mean steady state therapeutic levels of paracetamol (>10 mg/l). However, lower peak concentrations were seen after the first dose, which suggested the need for a 1500 mg loading dose in order to achieve a rapid onset of maximum therapeutic efficacy (Fig. 4). A fixed maintenance dose of 600 mg intramuscular or oral syrup every four hours also showed adequate steady state concentrations but this regimen failed to reach the therapeutic levels during the first 6 h after administration (Fig. 4).

The linear regression analysis showed no statistically significant relationship between C_MAX_ and temperature reduction two hours post-dose (*P* = 0.65). There was a trend of a longer parasite half-life with higher C_MAX_ but this was not statistically significant (*P* = 0.20). When stratified by administration route, the relationship between the intramuscular C_MAX_ and parasite clearance half-life was more evident (*P* = 0.030). However, the pharmacodynamic model assessing the relationship of paracetamol concentrations and the relative change in parasite clearance half-life showed no statistical improvement of adding paracetamol concentrations as a direct-effect on the estimated parasite clearance rate (ΔOFV = −2.85). Adding a hypothetical effect-compartment to account for a delayed effect of paracetamol on parasite clearance (hysteresis) showed no improvement of the model (ΔOFV = −0.06).

## Discussion

Although paracetamol has been used clinically as an antipyretic for over 100 years, there is a paucity of literature describing its pharmacokinetic properties after intramuscular administration. Paracetamol is by far the most widely used antipyretic in malaria, one of the most common causes of fever in tropical countries. Patients with malaria often vomit, particularly with high fever, and in cerebral malaria are unconscious, so the intramuscular route provides an alternative administration option (in the absence of a bleeding tendency). In this study the disposition of paracetamol was best described by a two-compartment disposition model, which is consistent with previous pharmacokinetic reports [27]. Since one-compartment structural models of oral syrup paracetamol pharmacokinetics have been reported, separate analyses of structural models for intramuscular and oral administration were performed [28]. The results showed that the two-compartment disposition of the final pharmacokinetic model was driven by the intramuscular data. A one-compartment disposition model was adequate for oral syrup administration demonstrating that the rapid distribution phase after parenteral administration was obscured by the oral absorption phase. As expected, the absorption of paracetamol administered intramuscularly and orally was best described by zero-order and first-order absorption, respectively. A more flexible transit absorption model did not result in a statistical improvement when fitting the absorption data after oral administration. This may be a consequence of few data in the absorption phase. The C_MAX_ was observed at approximately 40 min after both intramuscular and oral administration, which is also similar to previous reports [26, 29]. However, few data points in the absorption phase might bias these estimates and they should be interpreted with caution. The relative oral bioavailability compared to intramuscular administration was 84.4 % (95 % CI 68.2–95.1 %). This is in agreement with a previously reported absolute oral bioavailability of paracetamol syrup of 87 % [30]. This bioavailability of oral tablets of paracetamol is usually reported as slightly lower (i.e., 63–90 %), presumably because of better absorption of syrup formulation [31, 32]. The C_MAX_ of paracetamol after intramuscular and oral administration (600 mg) were 11.4 and 8.52 mg/l, respectively. The lower C_MAX_ of oral paracetamol is explained by incomplete absorption and the first-pass metabolism that occurs during absorption before paracetamol enters the systemic circulation, and the slower absorption obscuring distribution from an apparent central compartment [9]. While the pharmacokinetics of paracetamol in severe falciparum malaria have not been studied, the bioavailability of oral paracetamol may be even lower given the decrease in gastric emptying [33] and splanchnic blood flow [34] observed in severe malaria. Slower absorption of intramuscular paracetamol would also be expected in severe malaria. Although the bioavailability of intramuscular paracetamol is higher than the oral route, the relatively high cost of a 3 day course (1 g every 6 h) of parenteral paracetamol (intramuscular: 4.87 USD* (Atlantic Laboratory); intravenous: 53.28 USD* (generic Perfalgan) *excluding additional costs of administration) compared to oral paracetamol (tablets: 0.24 USD; syrup: 2.40 USD) [35] and the limited availability restrict the global use of parenteral paracetamol.

The population pharmacokinetic model of paracetamol showed large inter-individual variability in most pharmacokinetic parameters, probably due to small sample size and limited data for each route of administration. However, the visual predictive check of the final pharmacokinetic model suggested adequate predictive performance.

Dosing simulations of a 1500 mg loading dose followed by a maintenance dose of 1000 mg every 6 h resulted in more favourable paracetamol plasma concentration–time profiles, reaching maximum therapeutic concentrations rapidly after the first dose. This suggests that a loading dose might be needed for a rapid onset of maximum antipyretic effects. Dosing simulations of intramuscular and oral syrup paracetamol administered at a dose of 600 mg every 4 h showed that this regimen reached therapeutic steady state concentrations but with a delayed onset of action. However, the total daily dose of paracetamol that would be administered with the loading dose regimens would be 4.5 g/day, which is above the recommended maximum daily adult dose of 4 g.

Administration of tablets and suppositories may not reach therapeutic concentrations because of lower bioavailability compared to orally administered paracetamol syrup [15, 26, 36–38]. Thus, currently recommended dose regimens for tablets and suppositories may not be sufficient to reach similar steady-state concentrations compared to paracetamol syrup. Therefore, the limited effect of paracetamol on fever clearance reported in uncomplicated malaria studies that administer suppositories or tablets, or do not directly observe therapy, may reflect sub-therapeutic paracetamol levels [6, 39].

Paracetamol is potentially hepatotoxic and total adult doses over 4 g/day are not generally recommended. Although not used widely, high doses of paracetamol have been studied both as single loading doses and as multiple dosing regimens that exceed 4 g/day. A single 2 g dose of intravenous paracetamol for post-operative pain has been shown to be efficacious and safe in patients undergoing dental surgery compared to a 1 g dose [40]. A multiple dose regimen of 2 g intravenous paracetamol followed by 1 g every 6 h (total, 5 g in 24 h) in healthy subjects showed no drug accumulation during the regimen and no hepatotoxicity at 72 h after the first dose [41]. The mean C_MAX_ measured 15 min after the 2-g intravenous infusion was 67.9 ± 21.8 μg/ml, which is below the toxic range. Multiple-dose regimens of 6 g per day for 3 days (1 g orally every 4 h) studied in stroke patients showed a significant temperature lowering effect and no significant hepatotoxicity compared to placebo [42, 43]. In the current study, a simulated 1500 mg loading dose followed by 1000 mg every 6 h achieved therapeutic concentration–time profiles of paracetamol rapidly when administered by either route. Although the total daily dose of this regimen (4.5 g/day) exceeds the recommended maximum adult daily dose, the simulated maximum plasma concentrations were well below the potential hepatotoxic threshold concentration of 150 mg/l measured 4 h post-dosage [44]. Also, the bioavailability of the loading dose paracetamol regimen administered by intramuscular and oral syrup routes is expected to be lower than the intravenous (5 g/day) regimen used in other studies [41]. Evidently, larger clinical safety and efficacy assessments of this regimen would be required to confirm the general applicability of loading doses of paracetamol in this population.

Febrile temperatures have been shown to accelerate and increase cytoadherence of parasitized erythrocytes in vitro [3]. In a study of African children receiving regularly dosed rectal paracetamol, it was suggested that those receiving paracetamol had a prolonged parasite clearance time compared to patients treated with mechanical antipyresis [6]. One hypothesis is that paracetamol reduces fever, which may then result in less cytoadherence, sequestration and thus increase circulating peripheral blood parasitaemia. This effect could be interpreted as a prolonged parasite clearance time. The pharmacodynamic model in this study showed that higher paracetamol concentrations resulted in an insignificant temperature reduction, and there was a trend to prolongation of parasite clearance rate. The lack of a significant pharmacodynamic effect observed in this study is likely due to the single daily low dose of paracetamol administered and the small sample size.

The small sample size and limited number of pharmacokinetic samples are limitations of the current study. Larger studies are warranted to determine the safety and efficacy of the proposed loading dose regimen.

## Conclusions

The population pharmacokinetic properties of paracetamol were characterized after both intramuscular and oral syrup administration. Oral bioavailability was estimated as 84.4 % relative to intramuscular administration in patients with acute uncomplicated falciparum malaria. Modelling and simulation showed that 1 g of paracetamol administered six-hourly by intramuscular or oral syrup routes predicted therapeutic steady-state concentration–time profiles. Maximum therapeutic steady-state concentrations were reached more rapidly by adding a loading dose to the standard regimen. This pharmacokinetic analysis showed that sub-therapeutic paracetamol concentrations are reached after a single 600 mg dose of paracetamol, which may explain the insignificant temperature lowering effect of paracetamol observed in this study. The safety and efficacy of the loading dose regimens needs to be established in this population to ensure that adequate antipyretic dosing is administered to patients with uncomplicated falciparum malaria.
